# Evidence for Individual Differences in Behaviour and for Behavioural Syndromes in Adult Shelter Cats

**DOI:** 10.3390/ani10060962

**Published:** 2020-06-01

**Authors:** Sandra Martínez-Byer, Andrea Urrutia, Péter Szenczi, Robyn Hudson, Oxána Bánszegi

**Affiliations:** 1 Posgrado en Ciencias Biológicas, Unidad de Posgrado, Edificio A, 1er Piso, Circuito de Posgrados, Ciudad Universitaria, Coyoacán, CP 04510, Mexico; brownie_byer@ciencias.unam.mx (S.M.-B.); andreaurrutia@outlook.com (A.U.); 2 Instituto de Investigaciones Biomédicas, Universidad Nacional Autónoma de México, Mexico City, A.P. 70228, CP 04510, Mexico; rhudson@biomedicas.unam.mx; 3 CONACYT—Instituto Nacional de Psiquiatría Ramón de la Fuente Muñiz, Unidad Psicopatología y Desarrollo, Calz. México-Xochimilco 101, CP 14370, Mexico

**Keywords:** individual differences, behavioural assays, behavioural syndromes, companion animal, *Felis silvestris catus*, shelter cats, human-cat relation

## Abstract

**Simple Summary:**

An important activity of modern animal shelters is the development of successful adoption programmes. In this regard, there is a need for reliable tests of individual differences in behaviour to help match the “personality” of potential adoptees with the lifestyle and needs of prospective owners; a companion animal for an elderly person remaining at home requires a different match than a pet for someone who will be away most of the day; a pet kept exclusively indoors in a small apartment requires a different match than an indoor/outdoor pet. In the present study, we repeatedly tested 31 mixed-breed adult cats of both sexes and a wide range of ages in five behavioural tests at a shelter in Mexico City, Mexico. The tests were designed to be easily implemented by shelter staff, and were short and low cost and intended to simulate common situations in a pet cat’s everyday life. We found consistent (stable) individual differences in the cats’ behaviour on all five tests, as well as correlations between their behaviour across tests. This suggests that such tests may contribute to reliably characterizing the “personality” of individual cats and so help increase the rate of successful adoptions.

**Abstract:**

Consistent inter-individual differences in behaviour have been previously reported in adult shelter cats. In this study, we aimed to assess whether repeatable individual differences in behaviours exhibited by shelter cats in different situations were interrelated, forming behavioural syndromes. We tested 31 adult cats in five different behavioural tests, repeated three times each: a struggle test where an experimenter restrained the cat, a separation/confinement test where the cat spent 2 min in a pet carrier, a mouse test where the cat was presented with a live mouse in a jar, and two tests where the cat reacted to an unfamiliar human who remained either passive or actively approached the cat. Individual differences in behaviour were consistent (repeatable) across repeated trials for each of the tests. We also found associations between some of the behaviours shown in the different tests, several of which appeared to be due to differences in human-oriented behaviours. This study is the first to assess the presence of behavioural syndromes using repeated behavioural tests in different situations common in the daily life of a cat, and which may prove useful in improving the match between prospective owner and cat in shelter adoption programmes.

## 1. Introduction

For years, the domestic cat (*Felis silvestris catus*) has been among the most popular pets in the world [1,2]. Interest in cat behaviour, and particularly in inter-individual differences (animal personality), is reflected in recent reviews [3,4,5,6] and special issues in scientific journals treating such topics [7,8]. The cat is a good candidate for the study of individual differences as it is readily accessible and has a rich behavioural repertoire. It is also by far the most studied feline species in this respect [3]. As with other domestic animals (companion, farm and working animals), taking into account cats’ personality differences when rehoming or selecting them for specific tasks can have implications for management, welfare and economy [3,9,10]. 

Broadly defined, animal personality refers to relatively stable inter-individual differences in behaviour [11,12,13]. When several of these behaviours correlate across contexts, they can be characterized as a behavioural syndrome [12,14,15]. The most common methods used to study individual differences in behaviour in the cat include observation [16,17], owner surveys [18,19] and behavioural tests [20,21]. The latter have the advantage that they can be used to evaluate and quantify the stability of individual differences across repeated standardised testing. Since an individual’s behaviour is expected to be variable to some degree, some behaviours may be inconsistent and therefore less informative of the individual’s behaviour at a later time. Therefore, when testing cats, reliable methods are needed, i.e., behavioural tests and measures that have been found to be highly repeatable.

Many studies of cat personality or temperament are based on behavioural observation ([3,4] see reviews), which provide important information about cats’ behaviours in their daily environments. However, to explore cats’ reactions to specific situations, behavioural tests are necessary. The two most commonly used tests in cat personality research are novel object tests, where the animal is presented with an unfamiliar object, and tests of reaction to either familiar or unfamiliar humans [3]. Novel object tests tend to use stimuli of unclear biological relevance (e.g., a fan with paper streamers, a remote-control car, a metal container with a spring, or a wooden box; [20,22,23]). While these tests have been reported to reveal individual differences, their meaning in daily situations of the life of the cat is unclear. Therefore, in the present study, we decided to test the behavioural responses of cats to situations corresponding to what they would likely encounter in real-life situations.

Given cats’ popularity as companion animals, there has been a tendency to study their individuality in terms of their interaction with humans, for example, in their reaction to approach or handling by a familiar or unfamiliar person [20,24,25,26,27]. Other behaviours of interest for both companion and working cats (particularly mousers) include their reaction to everyday stressful situations or to prey, respectively. However, we are unaware of any studies that have experimentally addressed the inter-individual consistency of behavioural differences in these situations. Nevertheless, animal shelters have begun to implement personality testing as part of their adoption programmes, favouring a combination of surveys and behavioural testing, as in the Feline Temperament Profile [21] and the Meet Your Match Feline-ality assessment [28].

The present study is the first to incorporate repeated measurements using several behavioural tests and to take a behavioural syndrome approach by evaluating correlations among these measurements in a heterogeneous population of cats (wide age range, different backgrounds) housed in an animal shelter. Animal shelters have a continuing need for reliable personality tests, for example, to better match potential pets with prospective owners and households or to identify cats that may better fit a specific situation, such as working or therapy cats. We used five behavioural assays that we consider to be ethologically and ecologically relevant to the daily life of the domestic cat, repeated three times each (see details below). We previously reported an analysis of data which included a subset of the data presented in the present paper, gathered during the separation/confinement test [29], but here we include further behavioural tests with the aim of identifying a larger range of repeatable individual differences and behavioural syndromes.

## 2. Materials and Methods

### 2.1. Study Site and Animals

We collected data from 31 adult cats (14 males and 17 females) from a shelter in Mexico City, Mexico, aged between 1 and 11 years (mean 4.5, SD 2.6, Supplementary Material Table S1). In some cases, the cats’ ages were not known with certainty and were estimated by veterinarians. Participants were chosen randomly from among the cats at the shelter, which were in good health and permitted handling. All the cats had been neutered and had received post-operative care by qualified veterinarians within three days of entering the shelter, and all cats participating in the study had been at the shelter for at least six weeks prior to the start of behavioural testing. The shelter was a four-storey house divided into sections; approximately 50 cats were housed in each section according to how well they tolerated each other. All sections consisted of at least two rooms (approx. 2.5 × 3.5 m each) with access to a fenced outdoor area (approx. 2 × 4 m). Each cat was free to roam within its section. The rooms were furnished with cat beds, boxes of assorted sizes with blankets, scratchers and toys. Water, commercial dry cat food and sand boxes were always available.

### 2.2. Procedures

Tests were performed weekly for 12 sequential weeks; each of the five tests was performed three times across three sequential weeks (the human approach tests were performed on the same days). One test was performed per day on all subjects, tested in randomized order between 13:00 and 18:00. Not all cats were available for all trials, therefore sample sizes differ slightly between the tests (see Supplementary Material Table S1 for information on which cats participated in each test). All tests were video recorded (GoPro^©^ Hero3+, GoPro, Inc., San Mateo, CA, USA) for subsequent behavioural analysis.

### 2.3. Behavioural Testing

#### 2.3.1. Struggle Test

We proposed the struggle test as a proxy for the handling tests used in different mammalian [30,31,32,33] and bird species [34,35,36]. Since domestic cats are frequently handled by their owners, by other familiar and unfamiliar humans, and by veterinarians, we redesigned this test to evaluate the struggle response when they are picked up and restrained. We tested 30 adult cats (13 males and 17 females; mean age 4.5, SD 2.6 years, min = 1, max = 11). The test was performed in the section of the shelter where the cat normally resided. One of the experimenters (S.M.-B.) approached the cat and stroked it three times from the head to the base of the tail, then picked it up, holding it with both hands around the thorax, under its forelimbs. The test lasted until the cat began to struggle (see Table 1 for behavioural definition) or until 30 s elapsed after picking it up. When this happened, the cat was immediately set down. The experimenter wore gloves as a precaution against scratches. 

#### 2.3.2. Separation/Confinement Test

Separation/confinement tests are used for personality testing in many animals, particularly in social species [37,38,39,40,41]. Despite the fact that cats are considered only facultatively a social species [42,43], in previous studies this type of test has been successfully used for evaluating individual differences in kittens of the domestic cat [44,45] and adult shelter cats [29]. Moreover, this test represents a common situation in a cat’s daily life around humans, since cats are often confined in a carrier to take to other places outside their home.

The data from this test combined with other data from additional shelter cats have been previously reported in Urrutia et al. [29]. We tested 28 adult cats (12 males and 16 females; mean age 4.6, SD 2.7 years, min = 1, max = 11). Tests were performed in a small closed room unfamiliar to the cats; the room was 1.5 × 2 m, with flat-finished, unpainted concrete floor, walls and ceiling, and without furnishings. During the test, no other animals or humans were allowed to enter either the test room or the room adjacent to it to limit auditory and olfactory contact. One experimenter approached the cat (either S.M.-B. or A.U.), briefly stroked it and then carried it in her arms into the test room. With the help of a second experimenter, they placed the cat inside a standard commercial pet carrier (42 × 61 × 38 cm), which was a closed plastic box with a steel grill door at one end and ventilation holes along the sides. The carrier, with the cat inside, was then placed on the floor at a previously marked position and the experimenters left the room. The test lasted two minutes. Once this time had elapsed, the cat was removed from the carrier and returned by one of the experimenters to its home room. The video camera was set up 60 cm from the carrier. To improve visibility, a red light was mounted inside the carrier. The carrier was cleaned between trials with isopropyl alcohol. See Table 1 for definitions of the behaviours analysed in this test. 

#### 2.3.3. Mouse Test

In our experience, neither kittens nor adult cats show sustained interest in interacting with the types of inanimate objects conventionally used in novel object tests. We therefore chose tame, laboratory-strain (BALB/c) mice as the “novel object” to more closely approximate a biologically relevant stimulus, since small rodents are the most common prey of the domestic cat [46,47,48,49,50] and because of the ease with which they can be maintained and handled (see below for details on how the mouse was presented; see also [51]). In a previous study by Yang et al. [52], the BALB/c mouse strain was found to show the least fearful reactions in response to a predator. In our tests, a total of five mice were used in rotation; three of them were taken to the shelter on test days. The mouse in the jar was switched every two trials (approx. 10 min) to minimize stress. The stimulus animals showed no obvious signs of fear in the presence of the cats; there were no signs of panic (e.g., freezing) or attempted escape or defence (e.g., jumping), they moved around in the jar in apparent calm, sometimes adopting the stretch–attend posture—which according to previous research is indicative of risk assessment rather than a fearful reaction [52]—in apparent curiosity at the presence of the cats. At the end of the study, the mice were adopted by student participants. For more details on the housing of the mice outside the tests, see Supplementary Material File S2. Additionally, during pilot tests, thermal pictures of the mice were taken before and after being in the jar with a cat in the room. Analysis of these images showed that the stress experienced by the mice (as measured by the increase in eye temperature) was comparable to that experienced in routine laboratory tests [53,54].

We tested 23 adult cats (7 males and 16 females; mean age 4.4, SD 2.5 years, min = 1, max = 11). Cats were individually tested in an unfamiliar room (4 × 6 m) which was cleared of all other cats and any objects that could be distracting. Subjects were given a two-minute habituation period before introducing the mouse. During habituation, and throughout the test, an experimenter (S.M.-B.) remained in the room, standing motionless and silent in a corner.

At the end of the habituation period, the experimenter restrained the cat in the middle of the room while a second experimenter brought in a mouse inside a clear, thick glass jar (15 cm in diameter × 20 cm high) with a perforated lid and covered with a cardboard box. At a marked position approximately 1.5 m from the cat and against a wall, the second experimenter fixed the jar to the floor with double-sided tape, removed the cardboard box and left the room. The first experimenter then released the cat and returned to the corner. The cat could see and presumably hear and smell the mouse but could not access it. The cat was free to interact with the jar for two minutes, after which the test ended and the cat was returned to its section of the shelter. The video camera was mounted on the wall 2 m above the jar. See Table 1 for definitions of the behaviours analysed. 

#### 2.3.4. Human Approach Tests

Human approach tests have been commonly used to evaluate cat behaviour [20,25,55,56,57], especially in shelters [27,58]. We modified the test from Adamec et al. [59] and tested the response of 28 adult cats (11 males and 17 females; mean age 4.6, SD 2.7 years, min = 1, max = 11) to an unfamiliar person. This person, a male volunteer, was the same person on a given test day but a different volunteer each week (age 21–25 years). To minimize unintentional odour cues, all were non-cat owners, were asked to wear fresh clothes and were unknown to the cats. Thus, the cats had the opportunity to interact with three different humans, one in each trial.

- Passive human approach test

Tests were performed in the same room as described for the mouse test. Before testing, two concentric circles, 1.5 and 3 m in diameter, were drawn on the floor with chalk to use as references of cat–human distance in the later video analysis, and the male volunteer was asked to sit cross-legged on the floor in the centre of the inner circle. When the volunteer was in position, the cat was carried in arms into the room by a familiar experimenter and placed in a shallow (20 cm deep) open wooden box against the wall next to the door. The experimenter then left the room. The test started when the door closed, leaving the cat alone with the unfamiliar person. The test consisted of two parts. For the first three minutes the unfamiliar volunteer sat cross-legged on the floor, looking at the wall and ignoring the cat however close it got. We used an approach score from 1 to 5 depending on whether the cat did the following: (1) remained outside the large circle; (2) entered at least its forepaws in the large circle; (3) entered at least its forepaws in the small circle; (4) established physical contact with the human (rub, sniff, touch with paw); (5) put at least its forepaws on top of the human. Then, in the second part, the volunteer continuously called the cat by its name for one minute while extending his arm and index finger as a greeting, pointing in the cat’s direction, even if the cat had already made physical contact with him. See Table 1 for definitions of the behaviours analysed in this test.

- Active human approach test

This test was performed immediately after the passive human approach test. The volunteer was instructed to slowly rise to his feet, approach the cat and attempt to stroke it six times from the head to the base of the tail. If the cat moved away before it could be stroked six times, the unfamiliar human walked after it and attempted to stroke it again. The test ended after the sixth stroking attempt (whether successful or unsuccessful) or after 1 min. The experimenter then entered the room and returned the cat to its home room. 

### 2.4. Ethical Considerations

Throughout the study, animals were kept and treated according to the guidelines for the use of animals in research as published in Animal Behaviour (ABS, 2016), as well as the relevant legislation for Mexico (National Guide for the Production, Care and Use of Laboratory Animals, Norma Oficial Mexicana NOM-062-200-1999), and approved by the Institutional Committee for the Care and Use of Laboratory Animals (CICUAL, permission ID 6315) of the Institute of Biomedical Research, UNAM, Mexico City, Mexico.

### 2.5. Video and Statistical Analysis

All behavioural variables were coded using Solomon Coder software for video analysis [60]. Statistical analyses of the data were carried out using the programme R, version 3.6.1 (R Foundation for Statistical Computing, Vienna, Austria) [61]. Prior to fixed-effects and repeatability analyses, any non-normally distributed continuous variables were normalized using either a Box–Cox or log transformation with the R package MASS [62]. Effects of sex, age and trial number on behavioural variables were analysed using linear mixed-effects models (LMM) for continuous, and generalized linear mixed-effects models (GLMM) for count (i.e., Poisson distributed) or binary (binomially distributed) dependent variables with the R package lme4 [63]. As fixed effects, we included sex, trial number (1 to 3), age (as a covariate), the interaction of sex × age and the interaction of trial number × age. As a random factor, we included individual identity. We applied backwards stepwise reduction of the full models beginning with non-significant interactions followed by non-significant fixed effects when *p* > 0.05. Individual identity as a random factor was retained in all models to account for repeated measures of individuals. *p*-values were extracted by Wald chi-squared tests (type III). 

We then analysed the repeatability of individuals’ behaviour across the three trials by intra-class correlations calculated as the proportion of phenotypic variation that can be attributed to between-subject variation [64]. We used GLMM-based calculations for count (Poisson distributed) or binary (binomially distributed) data and LMM-based calculations (Gaussian distributed) for continuous data for testing the repeatability of individual differences using the R package rptR [65,66]. Individual identity was used as a random factor and the fixed effects found to have a significant effect on each behaviour in the previous analysis were included where applicable. For all intra-class correlations, we calculated 95% confidence intervals by 1000 bootstrap steps, and *p*-values were calculated by 1000 permutations.

To investigate the possible association of the behaviours between the different tests, we first performed principal component analyses (PCAs) independently on each of the following tests: separation/confinement, mouse and passive human approach using spectral decomposition assuming correlation matrices, to reduce the number of dimensions; no rotations were used. In the case of the struggle and active human approach tests, we used the raw behavioural data, since only one behaviour was coded in each of these two tests. Since phenotypic correlations between traits may originate from two sources, that is, (i) from individuals’ average levels of two traits (between-individual correlation) or (ii) from individuals’ change in behaviour (within-individual correlation) [67,68,69,70], we calculated between-individual and within-individual (residual) correlations by using multivariate linear mixed models with the R package sommer [71] to partition possible phenotypic correlations between the traits. *p*-values were corrected for multiple tests using the Benjamini–Hochberg method.

## 3. Results

### 3.1. Repeatability of Individual Differences within Tests

#### 3.1.1. Struggle Test

No effects of age, sex or trial or of the interaction between these were found on the latency to struggle (Supplementary Material Table S3). All cats (*n* = 30) struggled within the 30-s limit, with only one cat still held at 30 s on one occasion. Individual differences in the latency to struggle were significantly repeatable across the three trials (Table 2). 

#### 3.1.2. Separation/Confinement Test

Age and trial number (1–3) were found to have a small, significant effect on the number of vocalizations and the duration of motor activity; older cats vocalized less and moved less in the carrier, and both behaviours diminished in consecutive trials (Supplementary Material Table S3). In the case of latency to initiate motor activity, there was a significant but very small effect of sex, where males began motor activity slightly sooner. There was a small effect of the interaction between age and sex, where the latency to move was slightly higher in older males than in younger males. There was also a small effect of trial number, where latency to begin motor activity began slightly later in consecutive trials (Supplementary Material Table S3). Therefore, these significant fixed effects were included in the respective repeatability analyses. Individual differences in the latency to vocalize and the number of vocalizations emitted by the cats (*n* = 28) were highly repeatable. Duration of motor activity was also significantly repeatable, although the latency to locomote was not (Table 2).

#### 3.1.3. Mouse Test 

The sequence of trials was found to have an effect on the duration of interactions (cats interacted less with the mouse on the third trial than during the first two trials) and was thus added as a fixed effect in the analysis (Supplementary Material Table S3). No other variable showed an effect of age, trial number or sex or the interaction between them. We found highly repeatable individual differences in the latency to approach and the time cats (*n* = 24) spent near the mouse across trials. Variables associated with proximity to the mouse were likewise repeatable, such as the time spent walking around the jar, the latency to interact and the duration of interaction (Table 2). Even tail swishing, which was coded from any area of the room, showed repeatable individual differences, a possible sign of interest or arousal of the animal even from afar. 

#### 3.1.4. Human Approach Tests 

None of the behavioural variables measured in these tests was significantly affected by age, trial number or sex or the interaction between them (Supplementary Material Table S3).

- Passive human approach test

We found repeatable individual differences (*n* = 28) for all behavioural measures in both phases of the test across trials, that is, the distance individual cats kept from the unfamiliar human was consistent even though each of the three trials used a different unfamiliar volunteer. We also found repeatable individual differences for the finger-nose contact measure of phase two. Moreover, individual differences in the latency to vocalize and in the number of vocalizations emitted during the entirety of trials were also highly repeatable (Table 2).

- Active human approach test

Individual differences in the latency for the unfamiliar person to be able to stroke the cat were consistent across trials and even though this involved three different people (Table 2). 

### 3.2. Correlations Between Tests

For dimension reduction purposes, we performed three separate PCAs on the behavioural variables of the following tests: separation/confinement, mouse and passive human approach. For the full results of the PCAs, see Supplementary Material Table S4. In the separation/confinement test, two principal components were extracted. For factor 1 (“confinement/separation vocalization”), the behaviours with the highest loadings were those related to vocalization and, for factor 2 (“confinement/separation motor activity”), the highest loading was the duration of motor activity. In the mouse test, two principal components were extracted. For factor 1 (“interaction with the mouse”), the behaviours with the highest loadings were related to the cats’ proximity to and interaction with the mouse jar and, for factor 2 (“tail swishing”), the highest loading was for the duration of tail swishing. In the passive human approach test, two principal components were also extracted. For factor 1 (“approaching the passive human”), the behaviours with the highest loadings involved the human approach score and finger–nose contact; for factor 2 (“passive human approach vocalization”), the behaviour with the highest loading was the number of vocalizations.

**Figure 1:**
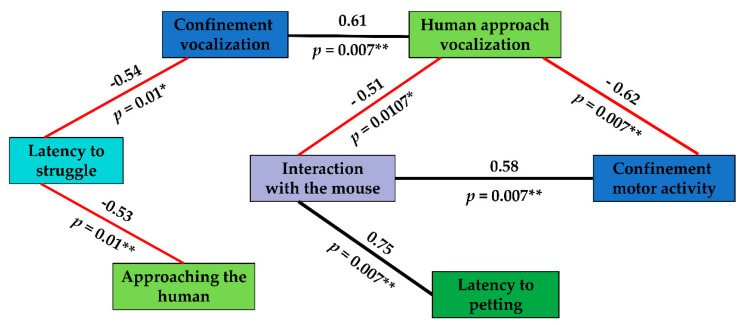
Correlations between behavioural variable scores showing stable individual differences at the between-individual level. Asterisks indicate significance levels at *p* < 0.05 *, *p* < 0.01 **. Black lines correspond to positive correlations, red lines correspond to negative correlations. Line thickness corresponds to the strength of a correlation. Further details are available in Supplementary Material Table S5, including confidence intervals.

In each of the two remaining tests (struggle and active human approach), we measured only one behavioural variable (latency to struggle and latency to be stroked by the human, respectively), hence we did not perform a PCA for these tests. Using the raw data for these variables, along with the six previously described factors obtained from the PCAs, we calculated correlations using multivariate linear mixed models. From a total of 34 correlations (Supplementary Material Table S5), we found eight that were significant after adjusting *p*-values for multiple comparisons (Benjamini–Hochberg method; Figure 1). 

## 4. Discussion

### 4.1. Consistency Across Time

In this study, we first evaluated the consistency across time of individual differences in behavioural responses of adult shelter cats in five different tests, and for all tests we found measures that showed significant repeatability. Stable individual differences were evident even though the cats were a heterogeneous population that differed in age, sex and (largely unknown) background. Perhaps surprisingly, individual differences in behavioural responses in most of the tests were unrelated to age or sex, suggesting that the behaviours measured here may be useful for evaluating individual differences in adult cats in general. This is supported by previous studies reporting stable individual differences in cats and other mammals in tests similar to those used here, that is, struggle or restraint tests used in cats [26], mice [31], rabbits [32,72,73], North American red squirrels [74,75] and pigs [33,76]; social separation tests used in cats [29,44,45], horses [77] cows [39,78] and dogs [40]; mouse tests used in cats [51,79]; and human approach tests used in cats [21,25,27,56,57,59], dogs [80], pigs and cattle [81,82]. These tests in their various forms are all relevant to the daily life of most cats, and thus provide a promising basis for assessing cat personality across a wide range of populations and conditions, including in shelter cats.

### 4.2. Behavioural Syndromes

We found seven significant correlations between behavioural scores from the different tests (Figure 1). Most of these seemed to be connected with humans; for example, cats that readily approached the unfamiliar human in the passive human approach test also struggled sooner in the struggle test, which may suggest that these cats were more confident around humans. Cats that struggled sooner also tended to vocalize (meow) more during the confinement test when separated from humans and other cats, suggesting that these individuals may seek the company of humans more, since meowing is considered a human-oriented behaviour ([83,84] our observation). Such correlations may indicate the existence of behavioural syndromes as defined in the Introduction. 

We can suppose that while the separation/confinement test was probably a negative experience for all the cats, the human approach test was a positive experience for at least some individuals. A more detailed acoustic analysis of the meows may help disentangle the emotional valence and motivation (e.g., stress, attention-seeking, greeting) underlying them in these tests, since meows emitted during distress have a distinct pattern (low mean fundamental frequency, longer duration; Schötz et al. [85]). Additionally, the cats for which the human approach test was a positive experience may have emitted other vocalizations (e.g., purrs, which Fermo et al. [86] found are exclusively associated with positive experiences). However, we were not able to record them due to their low volume. It is also possible, as Guillette and Sturdy [87] have suggested, that the degree of arousal or readiness of the cat to act (due to activation of the sympathetic nervous system) may contribute to the pattern of vocal emissions in different contexts [88]. 

Consistent with previous findings, we did not find an association between the number of vocalizations and motor activity within the confinement/separation test, suggesting different underlying mechanisms (motivation) between these variables (see more details in [29]). However, there was a negative correlation between motor activity in the confinement/separation test and the number of vocalizations emitted during the passive human approach test. The only explanation we can presently offer is that the cats for which the passive human approach test was a positive experience may have “carried” this correlation, meaning that possibly only positive meows are correlated with motor activity. Further study into the relationship between meows and motor activity in positive and negative situations may help to disentangle this.

Additionally, interaction with the mouse was significantly correlated with three different variables. It was negatively correlated with vocalization in the human approach passive phase, which can be interpreted as cats that were more focused on the mouse were less demanding of human attention (vocalized less). The latter is supported by the positive correlation between interaction with the mouse and latency to be stroked in the active human approach test, i.e., cats that spent more time with the mouse took longer to allow themselves to be stroked. Taken together, these correlations suggest a syndrome where more prey-oriented individuals are also less human-oriented. Although cats’ backgrounds in the present study were unknown, we speculate that such a syndrome may arise as a consequence of experiences prior to their arrival at the shelter, that is, cats that were more independent from humans may have relied more on hunting to obtain food, whereas cats that were more social with humans had relied on them for sustenance. Finally, there was also a positive correlation between interaction with the mouse and motor activity during the confinement/separation test, suggesting that some cats were more “excitable” than others, possibly due to differences in sympathetic nervous system arousal as discussed previously for vocalizations. 

### 4.3. Behavioural Testing in Animal Shelters

All five tests implemented in this study are simple and fast (no more than five minutes each), and any materials used are inexpensive and easily procured. Because of this, they can be reproduced practically anywhere in the world with minimal instruction of shelter personnel. Together, this makes them a suitable option for shelters looking to evaluate personality as part of their adoption programme. While millions of cats enter animal shelters every year, in the United States, for example, only an estimated 11.5% of pet cats come from a shelter [4,28]. Furthermore, even if a cat is adopted, there is still a high chance that it will be returned due to not fulfilling the new owner’s expectations, which risks euthanasia. Organizations like the American Society for the Prevention of Cruelty to Animals have managed to decrease the number of returned cats by applying questionnaires and personality tests [28]. 

However, these protocols are not applied worldwide, due to differences in owner expectations and the way shelters operate in different locations, among others. For example, animal shelter facilities in Mexico and throughout Latin America differ from those in the United States and Europe, something also noted by Fukimoto et al. [89] in their study of shelter cats in Brazil. Although our tests share some similarities with the ASPCA’s Feline-ality behavioural assessment, we sought to develop tests that could be a better fit for the shelter conditions and owner expectations we are familiar with. For example, we chose to use the pet carrier as a test within itself to evaluate individual responses to isolation and confinement, as separation anxiety is a common concern for owners who work long hours away from home. We also included a novel test (mouse test) in which the cats are presented with a biologically relevant stimulus. Although we recognize that this test will not be relevant to all cats that are offered for adoption as pets, nor is it feasible for all shelters to keep mice for this test, we would like to note that. In some shelters around the world. there are programmes to adopt out or loan “mouser” or “barn” cats ([c.f. [90] and also see the programs of the following organizations: Battersea Dogs & Cats Home (UK), Dereham Adoption Center (UK), Animal Humane Society (USA), Best Friends Animal Society (USA), Barn Cats Inc. (USA), among others). In recent years, there has been an increase in the demand for mousers by more environmentally friendly businesses and organic farms seeking to avoid rodenticides and to switch to biological pest control. This is an option for cats that are not sociable with people. Those individuals that show a strong interest in potential prey probably have a better chance of being successfully adopted into a working context.

Implementing repeated behavioural testing in the adoption process, whenever possible, could help match prospective owners with an animal that best suits the needs and lifestyle of both parties. For example, a family with small children needs a cat that tolerates handling; a calm person may want a calm cat; and someone who is not home most of the day would do better with a cat that is not stressed by separation. 

## 5. Conclusions

Reliable, economic and easily implemented behavioural tests are needed by animal shelters to improve their adoption programmes by improving the match between the personality of the prospective pet, in this case the cat, and the context of its new home. This can be best achieved by using tests based on the natural, evolved behaviour of the cat relevant to its everyday life and using correlations between more than one behavioural measure to form a more reliable profile of each individual cat’s personality. Results of the present study indicate that this is, indeed, feasible. 

## Figures and Tables

**Table 1:** Behavioural variables recorded in each test.

Behaviour Measured	Definition
**Struggle test**	
Struggle (latency)	Lifting one of the hind paws and touching or kicking the experimenter’s forearm
**Separation/confinement test**	
Vocalization (latency and number)	Meow-type vocalizations
Motor activity (latency and duration)	Displacement of any of the limbs on the floor or sides of the carrier for at least 1 s
**Mouse test**	
Near the mouse (latency and duration)	At least the front paws within 50 cm of the jar containing the mouse
Tail swishing (duration)	Any time the cat swished its tail from side to side at least twice
Interaction (latency and duration)	Contact with the jar, either sniffing or pawing
Walking around the jar (duration)	Walking from one side of the jar to the other while near it
**Passive human approach test**	
Approach score (1–5)	Maximum degree of proximity to the unfamiliar human
Vocalization (latency and number)	Meow-type vocalizations
Finger–nose contact (binary)	If the cat established contact by touching its nose to the human’s outstretched finger
**Active human approach test**	
Stroke (latency)	Latency to the first full stroke from head to tail in a set by the unfamiliar person

**Table 2:** Repeatability of the variables analysed for each of the behavioural tests. Intra-class correlation coefficients (*R*), 95% confidence intervals (lower bound, upper bound) based on 1000 bootstrap steps and significance values (*p*) are given. Asterisks indicate significance levels at *p* < 0.05 *, *p* < 0.01 **, *p* < 0.001 ***.

Behaviour	*R*	95% CI (lower bound, upper bound)	*p*-Value
**Struggle test**			
Latency to struggle	0.555	(0.314, 0.726)	0.001 ***
Separation/confinement test			
Latency to vocalize	0.761	(0.597, 0.861)	0.001 ***
Number of vocalizations	0.920	(0.766, 0.969)	0.006 **
Latency to motor activity	0.191	(0, 0.442)	0.066
Duration of motor activity	0.323	(0.06, 0.533)	0.001 ***
**Mouse test**			
Latency to approach	0.515	(0.248, 0.714)	0.001 ***
Duration near	0.498	(0.219, 0.679)	0.001 ***
Duration of tail swishing	0.806	(0.366, 0.944)	0.001 ***
Latency to interact	0.477	(0.201, 0.672)	0.001 ***
Duration interacting	0.501	(0.236, 0.716)	0.001 ***
Duration walking around	0.284	(0.019, 0.542)	0.017 *
**Passive human approach test**			
Approachscore (1–5)	0.312	(0, 0.507)	0.004 **
Latency to vocalize	0.668	(0.461, 0.806)	0.001 ***
Number of vocalizations	0.844	(0.632, 0.942)	0.008 **
Finger–nose contact (binary)	0.761	(0.376, 0.985)	0.001 ***
**Active human approach test**			
Stroking (latency)	0.496	(0.229, 0.692)	0.004 **

## Supplementary Materials

The following are available online at https://www.mdpi.com/2076-2615/10/6/962/s1, Table S1: Details of the subjects and test assignment, File S2: Description of the housing of the mice during the experiments, Table S3: Results of the LMMs and GLMMs to evaluate the impact of age, sex and trial number on behaviour, Table S4: Full results of the PCAs performed on the confinement/separation, mouse and passive human approach tests, Table S5: Correlations between behavioural variable scores showing stable individual differences.
