# Supplementary material for: Evidence for Individual Differences in Behaviour and for Behavioural Syndromes in Adult Shelter Cats
Source: Animals (Basel). 2020 Jun 1;10(6):962. doi: 10.3390/ani10060962 (PMC7341514; doi:10.3390/ani10060962)
Supplement: Supplementary file 1 [file animals-10-00962-s001.zip › Supplement/Supplement 1_cats info.docx]

**S1: Details of the subjects and test participation**

| **Name** | **Sex** | **Estimated age (years)** | Struggle test | Transport cage test | Mouse test | Human approach test |
| --- | --- | --- | --- | --- | --- | --- |
| Ani | F | 4 | X | X | X | X |
| Ani | F | 4 | X | X | X | X |
| Ann | F | 5 | X | X | X | X |
| Blanca | F | 9 | X | X | X | X |
| Casandra | F | 1 | X | X | X | X |
| Chipilina | F | 1 | X | X | X | X |
| Dora | F | 11 | X | X | X | X |
| Fekete | F | 4 |  |  |  | X |
| Ina | F | 4 | X | X | X | X |
| Johanna | F | 3 | X | X |  | X |
| Lilic | F | 3 | X |  | X | X |
| Lulu | F | 6 | X | X | X | X |
| Naomi | F | 7 | X | X | X | X |
| Nenia | F | 3 | X | X | X | X |
| Siberia | F | 7 | X | X | X | X |
| Squixi | F | 4 | X | X | X |  |
| Sutmi | F | 4 | X | X | X | X |
| Tsuki | F | 4 | X | X | X | X |
| Blue | M | 5 | X | X | X | X |
| Felix | M | 4 | X | X | X | X |
| Grisino | M | 4.5 | X | X | X | X |
| Guiseppe | M | 4 | X | X | X | X |
| Lenin | M | 1 | X | X | X | X |
| Lim | M | 2 | X | X |  | X |
| Munch | M | 6 | X | X | X | X |
| Obit | M | 8 | X | X |  | X |
| Ollin | M | 4 | X | X |  | X |
| Orange | M | 2 | X | X |  | X |
| Skit | M | 3.5 | X |  | X |  |
| Tigre | M | 9 | X | X |  | X |
| Tiqueri | M | 4 | X | X |  |  |
