# Supplementary material for: Evidence for Individual Differences in Behaviour and for Behavioural Syndromes in Adult Shelter Cats
Source: Animals (Basel). 2020 Jun 1;10(6):962. doi: 10.3390/ani10060962 (PMC7341514; doi:10.3390/ani10060962)
Supplement: Supplementary file 1 [file animals-10-00962-s001.zip › Supplement/Supplement 2_Mice.docx]

**S2: Description of housing of the mice during the study**

A total of five mice was used in rotation; three of them were taken to the shelter on test days. The mouse in the jar was switched every two trials (approx. 10 min) to minimize stress. While at the shelter the mice were kept in a large plastic container (23 cm x 27 cm x 32 cm high) containing wood shavings, cardboard tubes, food pellets and various fruit and vegetable pieces with a high-water content. When they were not at the shelter the mice were kept at a private home in a standard large laboratory cage (50 x 40 x 19 cm) lined with wood shavings and containing cardboard tubes and boxes; the mice had ad libitum access to food and water. The stimulus animals showed no obvious signs of fear in the presence of the cats; no signs of panic or attempted escape, moved around in apparent calm, reared against the front of the jar – which is considered as risk assessment – in apparent curiosity at the presence of the cats. Thermal pictures of the mice were taken before and after being in the jar with a cat in the room on several occasions. Analysis of the images with Fluke Smart View software showed that the maximum eye temperature increase was 1 °C, similar to the change in temperature reported during an open field test (Lecorps et al. 2016) and a control situation (Gjendal et al. 2018). Therefore, according to this physiological measure, the stress experienced by the mice in this test was comparable to that experienced in routine laboratory tests.

Gjendal K., Franco N.H., Ottesen J.L., Sørensen D.B., Olsson I.A.S. Eye, body or tail? Thermography as a measure of stress in mice. *Physiol. Behav.* **2018**, 196, 135-143. doi:10.1016/j.physbeh.2018.08.022.

Lecorps B., Rödel H.G., Féron C. Assessment of anxiety in open field and elevated plus maze using infrared thermography. *Physiol. Behav.* **2016**, 157, 209-216. doi:10.1016/j.physbeh.2016.02.014.
