# Supplementary material for: Evidence for Individual Differences in Behaviour and for Behavioural Syndromes in Adult Shelter Cats
Source: Animals (Basel). 2020 Jun 1;10(6):962. doi: 10.3390/ani10060962 (PMC7341514; doi:10.3390/ani10060962)
Supplement: Supplementary file 1 [file animals-10-00962-s001.zip › Supplement/Supplement 3_fixed effects.docx]

**S3: Effect of age, sex and trial number on behaviours**

﻿Bolded effects were significant and were thus later included in the repeatability analysis. Asterisks indicate signiﬁcance levels at *p <* 0.05 *, *p <* 0.01 **, *p <* 0.001 ***.

| Test / behaviour | Variable | Estimate | SE | *p* |
| --- | --- | --- | --- | --- |
| Struggle Test |  |  |  |  |
| Struggle (latency) | Age:sex (male) | -0.01 | 0.11 | 0.92 |
|  | Sex:trial | -0.16 | 0.14 | 0.27 |
|  | Trial | -0.01 | 0.07 | 0.84 |
|  | Sex (male) | -0.19 | 0.25 | 0.46 |
|  | Age | 0.07 | 0.05 | 0.21 |
|  |  |  |  |  |
| Separation / confinement Test |  |  |  |  |
| Vocalization (latency) | Age:sex (male) | 0.08 | 0.35 | 0.83 |
|  | Sex:trial | 0.14 | 0.34 | 0.68 |
|  | Trial | 0.04 | 0.17 | 0.82 |
|  | Sex (male) | -0.51 | 0.88 | 0.57 |
|  | Age | 0.32 | 0.17 | 0.07 |
|  |  |  |  |  |
| Vocalization (number) | **Age** | **-0.51** | **0.24** | **0.03 *** |
|  | **Trial** | **-0.16** | **0.05** | **0.001 **** |
|  | Age:sex (male) | 0.00 | 0.47 | 0.99 |
|  | Sex (male):trial | -0.09 | 0.11 | 0.39 |
|  | Sex (male) | -0.45 | 1.07 | 0.67 |
|  |  |  |  |  |
| Motor activity (latency) | **Age:sex (male)** | **0.02** | **0.01** | **0.04** * |
|  | **Trial** | **0.03** | **0.01** | **0.009** ** |
|  | **Sex (male)** | **-0.11** | **0.05** | **0.04 *** |
|  | Age | 0.004 | 0.007 | 0.59 |
|  | Sex (male):trial | 0.002 | 0.02 | 0.95 |
|  |  |  |  |  |
| Motor activity (duration) | **Trial** | **-0.52** | **0.08** | **<0.0001 ***** |
|  | **Age** | **-0.10** | **0.05** | **0.049 *** |
|  | Sex (male):trial | 0.14 | 0.16 | 0.37 |
|  | Age:sex (male) | -0.13 | 0.10 | 0.20 |
|  | Sex (male) | 0.21 | 0.25 | 0.41 |
|  |  |  |  |  |

(continued from previous page)

| Test / behaviour | Variable | Estimate | SE | *p* |
| --- | --- | --- | --- | --- |
| Mouse Test |  |  |  |  |
| Near the mouse (latency) | Age:sex (male) | 0.19 | 0.38 | 0.63 |
|  | Sex (male):trial | 0.43 | 0.42 | 0.30 |
|  | Sex (male) | 0.54 | 0.72 | 0.47 |
|  | Age | 0.14 | 0.14 | 0.31 |
|  | Trial | -0.34 | 0.20 | 0.10 |
|  |  |  |  |  |
| Near the mouse (duration) | Age:sex (male) | -0.21 | 0.42 | 0.63 |
|  | Sex (male):trial | -0.56 | 0.47 | 0.25 |
|  | Trial | -0.15 | 0.23 | 0.52 |
|  | Sex (male) | -0.88 | 0.80 | 0.29 |
|  | Age | -0.16 | 0.15 | 0.31 |
|  |  |  |  |  |
| Tail swishing (duration) | Age:sex (male) | -0.22 | 0.95 | 0.82 |
|  | Sex (male):trial | -0.45 | 0.95 | 0.64 |
|  | Trial | -0.10 | 0.45 | 0.82 |
|  | Sex (male) | 0.42 | 1.80 | 0.82 |
|  | Age | 0.37 | 0.33 | 0.28 |
|  |  |  |  |  |
| Interaction (latency) | Age:sex (male) | 0.02 | 0.06 | 0.77 |
|  | Sex (male):trial | -0.03 | 0.07 | 0.72 |
|  | Sex (male) | -0.01 | 0.12 | 0.93 |
|  | Trial | 0.00 | 0.03 | 0.90 |
|  | Age | 0.01 | 0.02 | 0.53 |
|  |  |  |  |  |
| Interaction (duration) | **Trial** | **-0.15** | **0.06** | **0.017 *** |
|  | Age:sex (male) | 0.03 | 0.11 | 0.83 |
|  | Sex (male):trial | -0.09 | 0.13 | 0.48 |
|  | Sex (male) | 0.06 | 0.22 | 0.80 |
|  | Age | -0.04 | 0.04 | 0.36 |
|  |  |  |  |  |
| Walking around the jar (duration) | Sex (male):trial | -0.20 | 0.99 | 0.85 |
|  | Age:sex (male) | -0.16 | 0.60 | 0.80 |
|  | Trial | 0.00 | 0.47 | 0.99 |
|  | Sex (male) | -0.87 | 1.14 | 0.46 |
|  | Age | -0.44 | 0.21 | 0.05 |
|  |  |  |  |  |

(continued from previous page)

| Test / behaviour | Variable | Estimate | SE | *p* |
| --- | --- | --- | --- | --- |
| Passive human approach Test |  |  |  |  |
| Approach score (1-5) | Age:sex (male) | -0.04 | 0.08 | 0.65 |
|  | Sex (male):trial | 0.08 | 0.17 | 0.65 |
|  | Trial | -0.05 | 0.08 | 0.56 |
|  | Age | 0.03 | 0.04 | 0.40 |
|  | Sex (male) | -0.24 | 0.21 | 0.24 |
|  |  |  |  |  |
| Vocalization (latency) | Sex (male):trial | -0.04 | 0.40 | 0.92 |
|  | Age:sex (male) | -0.10 | 0.37 | 0.79 |
|  | Sex (male) | 0.01 | 0.91 | 0.99 |
|  | Age | -0.04 | 0.17 | 0.81 |
|  | Trial | 0.13 | 0.20 | 0.52 |
|  |  |  |  |  |
| Vocalization (number) | Age:sex (male) | -0.10 | 0.40 | 0.79 |
|  | Sex (male):trial | 0.18 | 0.10 | 0.06 |
|  | Sex (male) | -0.10 | 1.00 | 0.92 |
|  | Age | -0.17 | 0.19 | 0.38 |
|  | Trial | -0.08 | 0.05 | 0.09 |
|  |  |  |  |  |
| Finger-nose contact (binary) | Sex (male):trial | 0.13 | 0.88 | 0.88 |
|  | Age:sex (male) | -0.17 | 0.68 | 0.80 |
|  | Age | -0.05 | 0.32 | 0.88 |
|  | Sex (male) | -1.22 | 1.71 | 0.47 |
|  | Trial | -0.54 | 0.44 | 0.22 |
|  |  |  |  |  |
| Active human approach Test |  |  |  |  |
| Stroke (latency) | **Trial** | **3.49** | **1.12** | **0.003 **** |
|  | Age:sex (male) | -0.41 | 1.46 | 0.78 |
|  | Sex (male):trial | 1.28 | 2.28 | 0.58 |
|  | Sex (male) | 3.77 | 3.62 | 0.31 |
|  | Age | -1.24 | 0.70 | 0.09 |
|  |  |  |  |  |
