# Supplementary material for: Evidence for Individual Differences in Behaviour and for Behavioural Syndromes in Adult Shelter Cats
Source: Animals (Basel). 2020 Jun 1;10(6):962. doi: 10.3390/ani10060962 (PMC7341514; doi:10.3390/ani10060962)
Supplement: Supplementary file 1 [file animals-10-00962-s001.zip › Supplement/Supplement 4_PCA.docx]

**S4:** **Full results of the PCAs performed on the confinement / separation, mouse and passive human approach tests**

In each case, separate PCAs were performed for each of the three trials to avoid pseudoreplication due to the repeated measures.

| **Separation / confinement test** |  |  |  |
| --- | --- | --- | --- |
| Factor 1 | Trial 1 | Trial 2 | Trial 3 |
| Eigenvalue | 1.72 | 1.74 | 1.99 |
| % variance | 57.3 | 58.1 | 66.4 |
| **Number of vocalizations** | **0.91** | **0.86** | **0.92** |
| **Latency to vocalize** | **-0.88** | **-0.89** | **-0.85** |
| Duration of motor activity | 0.34 | 0.45 | 0.66 |
|  |  |  |  |
| Factor 2 | Trial 1 | Trial 2 | Trial 3 |
| Eigenvalue | 0.96 | 0.91 | 0.75 |
| % variance | 32 | 30.3 | 25.1 |
| **Duration of motor activity** | **0.94** | **0.89** | **0.74** |
| Latency to vocalize | 0.28 | 0.17 | 0.43 |
| Number of vocalizations | -0.08 | -0.29 | -0.14 |

| **Mouse test** |  | |  |
| --- | --- | --- | --- |
| Factor 1 | Trial 1 | Trial 2 | Trial 3 |
| Eigenvalue | 3.16 | 3.49 | 3.06 |
| % variance | 52.7 | 58.2 | 51.1 |
| **Latency to be near** | **-0.94** | **-0.88** | **-0.91** |
| **Duration near** | **0.83** | **0.84** | **0.75** |
| **Latency to interact** | **-0.83** | **-0.83** | **-0.81** |
| **Duration interacting** | **0.81** | **0.92** | **0.85** |
| Duration of tail swishing | 0.36 | -0.01 | 0.21 |
| Duration walking around | 0.35 | 0.68 | 0.71 |
|  |  |  |  |
| Factor 2 | Trial 1 | Trial 2 | Trial 3 |
| Eigenvalue | 1.35 | 1.33 | 1.27 |
| % variance | 22.5 | 22.2 | 21.1 |
| **Duration of tail swishing** | **0.82** | **0.92** | **0.89** |
| Duration walking around | -0.65 | -0.46 | -0.43 |
| Duration near | 0.41 | -0.22 | -0.39 |
| Duration interacting | -0.25 | -0.06 | -0.11 |
| Latency to be near | 0.15 | -0.31 | -0.17 |
| Latency to interact | 0.07 | -0.36 | -0.33 |

| **Passive human approach test** |  | |  |
| --- | --- | --- | --- |
| Factor 1 | Trial 1 | Trial 2 | Trial 3 |
| Eigenvalue | 1.43 | 1.51 | 1.75 |
| % variance | 47.5 | 50.3 | 58.3 |
| **Finger-nose (0/1)** | **0.85** | **0.79** | **0.94** |
| **Human score** | **0.84** | **0.86** | **0.92** |
| Number of vocalizations | -0.03 | 0.39 | -0.15 |
|  |  |  |  |
| Factor 2 | Trial 1 | Trial 2 | Trial 3 |
| Eigenvalue | 1 | 0.98 | 1 |
| % variance | 33.4 | 32.7 | 33.4 |
| **Number of vocalizations** | **1** | **0.9** | **0.99** |
| Human score | 0.08 | -0.03 | 0.17 |
| Finger-nose (0/1) | -0.04 | -0.41 | -0.01 |
