# Supplementary material for: Evidence for Individual Differences in Behaviour and for Behavioural Syndromes in Adult Shelter Cats
Source: Animals (Basel). 2020 Jun 1;10(6):962. doi: 10.3390/ani10060962 (PMC7341514; doi:10.3390/ani10060962)
Supplement: Supplementary file 1 [file animals-10-00962-s001.zip › Supplement/Supplement 5_Correlations.docx]

**S5: Correlations between behavioural variable scores showing stable individual diﬀerences**

Table S5-1. Between-individual level behavioural correlations. *P*-values were adjusted for multiple comparisons using the Benjamini-Hochberg procedure. Significant correlations are bolded. Asterisks indicate signiﬁcance levels at *p <* 0.05 *, *p <* 0.01 **, *p <* 0.001 ***.

|  |  | ^+^Confinement / separation vocalization | ^+^Confinement / separation locomotion | ^+^Interaction with the mouse | ^+^Tail swishing with the mouse | ^+^Approaching the passive human | ^+^Passive human approach vocalization | Latency to petting success |
| --- | --- | --- | --- | --- | --- | --- | --- | --- |
| Latency to struggle | CI 95%  *p* | **-0.54**  **[-0.73, 0.26]**  ***p* = 0.01**** | 0.02  [-0.40, 0.44]  *p* = 0.94 | 0.25  [-0.26, 0.66]  *p* = 0.37 | 0.35  [-0.1, 0.68]  P = 0.17 | **-0.53**  **[-0.73, -0.26]**  **P = 0.01**** | 0.13  [-0.29, 0.51]  P = 0.704 | -0.12  [-0.54, 0.34]  P = 0.70 |
| ^+^Confinement / separation vocalization | CI 95%  *p* | - | 0.02  [-0.03, 0.07]  *p* = 0.95 | -0.16  [-0.54, 0.27]  *p* = 0.646 | 0.13  [-0.28, 0.5]  *p* = 0.704 | 0.34  [0.02, 0.6]  *p* = 0.169 | **-0.61**  **[-0.75, -0.42]**  ***p* = 0.007**** | 0.12  [-0.29, 0.49]  *p* = 0.704 |
| ^+^Confinement / separation locomotion | CI 95%  *p* | - | - | **0.58**  **[0.26, 0.78]**  ***p* = 0.007**** | -0.1  [-0.5, 0.34]  *p* = 0.704 | 0.29  [-0.08, 0.59]  *p* = 0.292 | **0.62**  **[0.42, 0.76]**  ***p* = 0.007**** | 0.41  [0.02, 0.69]  *p* = 0.072 |
| ^+^Interaction with the mouse | CI 95%  *p* | - | - | - | 0.17  [-0.06, 0.38]  *p* = 0.646 | 0.1  [-0.35, 0.51]  *p* = 0.704 | **0.50**  **[0.18, 0.73]**  ***p* = 0.017*** | **0.75**  **[0.57, 0.86]**  ***p* = 0.007**** |
| ^+^Tail swishing with the mouse | CI 95%  *p* | - | - | - | - | 0.11  [-0.31, 0.49]  *p* = 0.704 | 0.34  [-0.04, 0.63]  *p* = 0.169 | -0.23  [-0.6, 0.21]  *p* = 0.420 |
| ^+^Approaching the passive human | CI 95%  *p* | - | - | - | - | - | -0.07  [-0.42, 0.31]  *p* = 0.782 | -0.26  [-0.58, 0.12]  *p* = 0.357 |
| ^+^Passive human approach vocalization | CI 95%  *p* | - | - | - | - | - | - | -0.16  [0.24, -0.53]  *p* = 0.646 |

Table S5-2. Phenotypic and within-individual level behavioural correlations. Note that these correlations were only calculated in cases where more than one behavioural variable was measured in one test. Behavioural variables obtained via PCA are denoted with the symbol: ^+^. *P*-values were adjusted for multiple comparisons using the Benjamini-Hochberg procedure.

|  | Correlation type | ^+^Confinement / separation locomotion | ^+^Tail swishing with the mouse | ^+^Passive human approach vocalization | Latency to petting success |
| --- | --- | --- | --- | --- | --- |
| ^+^Confinement / separation vocalization | Phenotypic | 0.007  [-0.03, 0.04]  *p* = 0.972 | - | - | - |
|  | Within-individual | -0.03  [-0.06, -0.001]  *p* = 0.996 | - | - | - |
| ^+^Interaction with the mouse | Phenotypic |  | 0.01  [-0.13, 0.14]  *p* = 0.963 | - | - |
|  | Within-individual |  | -0.19  [-0.29, -0.08]  *p* = 0.996 | - | - |
| ^+^Approaching the passive human | Phenotypic |  |  | -0.01  [-0.26, 0.24]  *p* = 0.951 | -0.21  [-0.41, 0.01]  *p* = 0.816 |
|  | Within-individual |  |  | 0.09  [-0.13, 0.3]  *p* = 996 | -0.15  [-0.35, 0.07]  *p* = 996 |
| ^+^Passive human approach vocalization | Phenotypic |  |  |  | -0.09  [-0.31, 0.14]  *p* = 0.951 |
|  | Within-individual |  |  |  | 0.001  [-0.21, 0.22]  *p* = 996 |
